# A Rare Case Report of Acute Neurologic Sequelae in a Young Primigravida With Recent COVID Pneumonia

**DOI:** 10.7759/cureus.37520

**Published:** 2023-04-13

**Authors:** Sarah Hmaidan, Lynsa Nguyen, Andrea Johnson

**Affiliations:** 1 Obstetrics and Gynecology, Vanderbilt University Medical Center, Nashville, USA; 2 Obstetrics and Gynaecology, Vanderbilt University Medical Center, Nashville, USA

**Keywords:** neuro-covid, high-risk pregnancy, pediatric stroke, covid-19 in pregnancy, covid-19

## Abstract

Post-infectious neurological sequelae, particularly in the pediatric population, are a rarely observed and under-explored complication of COVID-19. Few case reports exist detailing severe neurological sequelae following acute infection with COVID-19, such as encephalopathy, stroke, and coma. This case report details the diagnosis and treatment of a 16-year-old primigravida with no past medical history who presented to the emergency department with rhythmic tremors, urinary incontinence, and generalized weakness two weeks following initial COVID-19 diagnosis with admission for pneumonia and sepsis. Vital signs were remarkable for tachycardia and normotension. Shortly following admission, she experienced generalized tonic-clonic seizure activity. Neurologic evaluation was remarkable for electroencephalogram with frontally predominant generalized periodic discharges and magnetic resonance imaging of the head showing bilateral parafalcine restricted diffusion. Cerebrospinal fluid analysis and magnetic resonance imaging of the spine were unremarkable. The patient was ultimately diagnosed with reversible cerebral vasoconstriction syndrome and an anterior cerebral artery stroke. Throughout the patient's recovery, she demonstrated incoherent, delirious, and disinhibited behavior that resolved within several days. She was ultimately discharged to a skilled rehabilitation facility with follow-up in a neurology clinic.

## Introduction

The outbreak of Coronavirus Disease 2019 (COVID-19) has led to a global pandemic that continues to affect millions of people and has overwhelmed virtually all hospital systems. Since the pandemic's inception, COVID-19 has been primarily transmitted via respiratory droplets. Symptoms are most often mild and commonly include fever and cough. Emerging reports have demonstrated increasing evidence of neurologic sequelae associated with COVID-19. A cohort study, including over 3700 patients, noted that approximately 80% of patients hospitalized with COVID-19 experienced neurologic manifestations, most commonly headache, anosmia, or ageusia [1]. More severe neurologic complications reported include encephalopathy, stroke, and coma [1]. Due to the rising number of people infected with COVID-19, pregnant patients and clinicians must be aware of the complications that can lead to rarer sequelae, specifically those that may present seemingly remote from the initial infection. Herein, we describe a case of a sixteen-year-old primigravida who presented with weakness, tremors, generalized tonic-clonic seizures, and altered mental status fourteen days following her initial admission for COVID-19-associated pneumonia and sepsis during pregnancy.

## Case presentation

**Figure 1:**
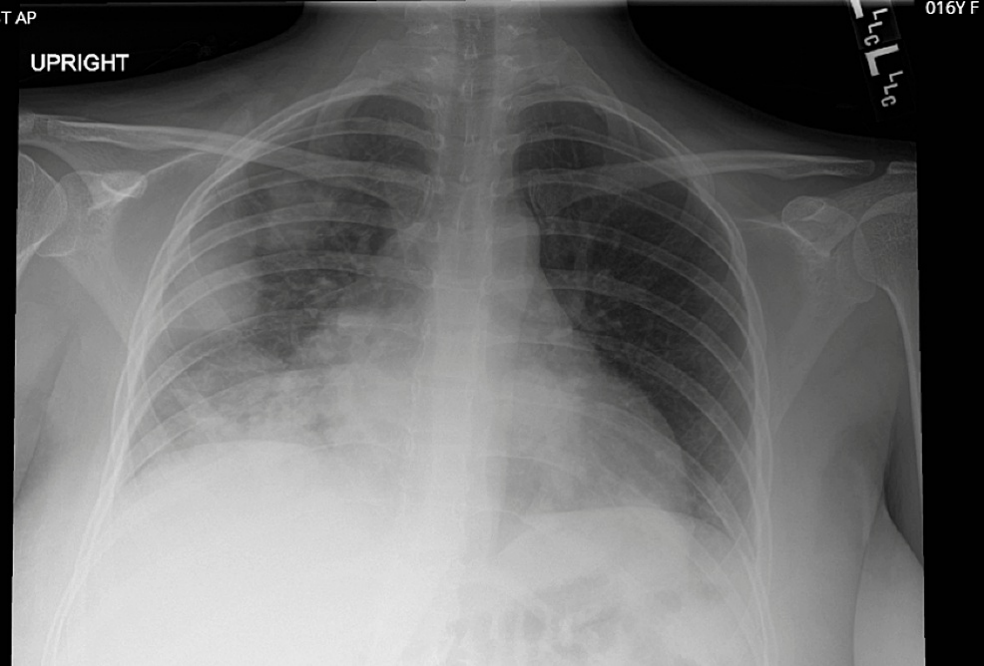
Chest X-Ray demonstrating patchy pulmonary opacities throughout the lungs, right greater than left.

A previously healthy sixteen-year-old primigravida unvaccinated against COVID-19 presented to an outside facility due to epigastric pain. During the workup, she tested positive for COVID-19 via a polymerase chain reaction test and was found to be incidentally pregnant. She had no pertinent medical, family, or social history. Ultrasound performed noted gestational age at 30 weeks 5 days. Later that day, she was transported to our facility, a level-4 trauma center in a private setting, and on arrival, was found to be in septic shock. She was febrile, hypotensive, tachycardic, and hypoxic. She was immediately transferred to the Intensive Care Unit (ICU) due to oxygen and pressor requirements. At the time of transfer, she required 100% non-rebreather. The Radiograph of the chest (Figure 1) demonstrated patchy pulmonary opacities throughout the lungs, the right greater than the left. A computed tomography angiogram (CTA) of the chest, completed to rule out pulmonary embolism, was negative. She was diagnosed with COVID-19 infection with superimposed bacterial pneumonia. In addition to supportive care, broad-spectrum antibiotics, dexamethasone, and remdesivir were initiated per hospital protocol for COVID-19 pneumonia. She remained in the SICU for four days and was ultimately transferred to the antepartum unit for further recovery. She was discharged after seven days in stable condition with the remaining course of dexamethasone and a close follow-up with an obstetrics provider.

**Figure 2:**
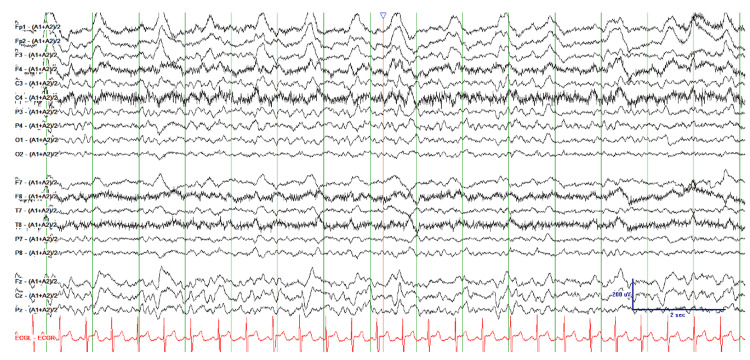
electroencephalogram with periodic discharges

The patient was re-presented to the emergency room fourteen days after discharge with new onset tremors of the right upper and lower extremities, urinary incontinence, and generalized weakness. Clinical examination revealed tachycardia to 150, a blood pressure of 117/89 mmHg, a respiratory rate of 21 breaths per minute, oxygen saturation of > 92% on room air, and a temperature of 98.5℉. She was placed on telemetry due to sustained tachycardia, which mildly improved after intravenous (IV) fluid administration. She subsequently experienced a generalized tonic-clonic seizure activity lasting approximately 90 seconds. She was immediately given 2mg IV Ativan, which aborted the seizure activity and initiated magnesium sulfate due to concern for eclampsia. However, she remained normotensive without other indicators of hypertensive disorders during pregnancy. Magnesium sulfate prophylaxis was discontinued, and Neurology was consulted. On exam, bilateral extremity weakness (4/5) and hyperreflexia (4+) were noted. Electroencephalogram (EEG) demonstrated frontally predominant generalized periodic discharges and left hemispheric voltage attenuation (Figure 2). She was started on levetiracetam due to concerns about seizures.

**Figure 3:**
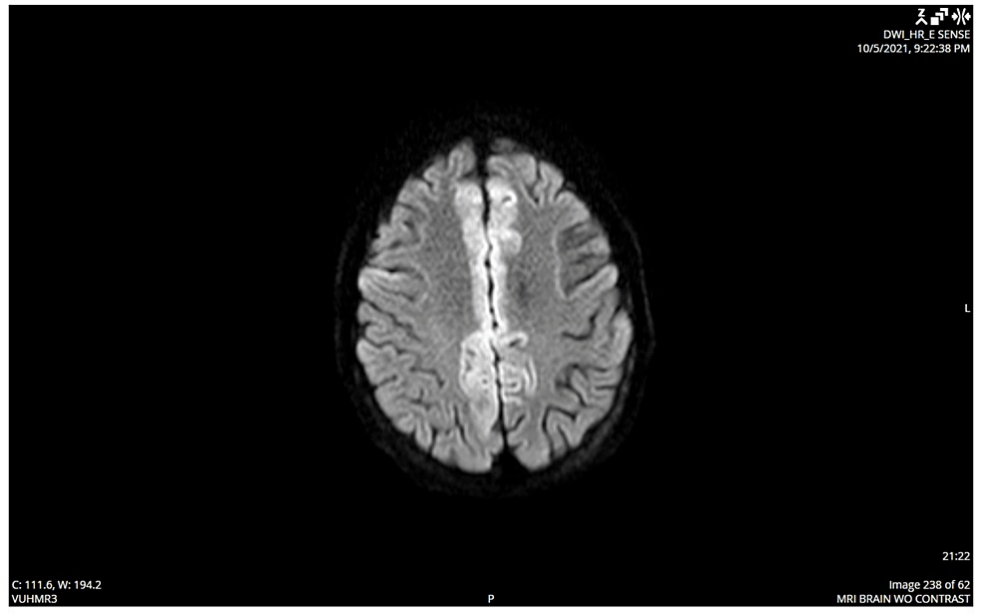
MRI brain without contrast with bilateral parafalcine-restricted diffusion

Head MRI (figure 3) showed bilateral parafalcine restricted diffusion; spine MRI without contrast was non-contributory. She was ultimately transferred to the ICU due to tachycardia, dysphagia, ascending bilateral weakness, and myoclonus. Workup for her persistent tachycardia included CT pulmonary angiogram and transthoracic echocardiogram, which were both unremarkable. She was intubated for airway protection due to progressive dysphagia and dysarthria.

**Figure 4:**
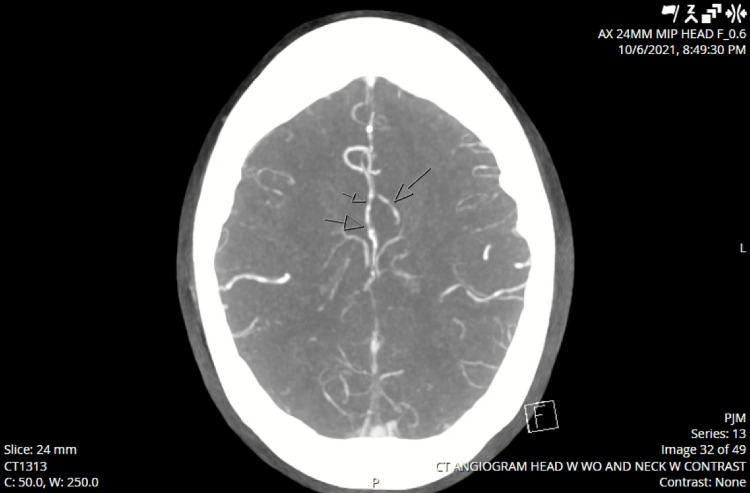
Diffusion-weighted MRI with bilateral anterior cerebral artery (ACA) distribution diffusion restriction

Although the patient continued to experience ascending weakness, concern for Guillain-Barre syndrome remained low due to brisk reflexes. Admission laboratory and cerebrospinal fluid studies were unremarkable overall (Table 1, 2). Blood cultures showed no growth, and a transthoracic echocardiogram was performed and did not demonstrate any abnormalities. Diffusion-weighted MRI on hospital day 6 demonstrated bilateral anterior cerebral artery (ACA) distribution diffusion restriction with new areas of restriction cortically in the insula with concurrent concern for an ACA infarct, correlating with the patient's bilateral motor weakness (Figure 4). Due to non-reassuring fetal status that day, she was delivered via urgent cesarean section. Intraoperatively, there was no evidence of chorioamnionitis or premature rupture of membranes.

**Table 1:** Admission Laboratory Studies CRP: c-reactive protein; TSH: thyroid-stimulating hormone

Laboratory Studies	Result	Reference Range
White Blood Cell Count	11.0 x 10^6^/mcL	3.4-10.2 x 10^6^/mcL
Hemoglobin	8.9 gm/dL	12.0-16.0 gm/dL
Hematocrit	28%	35-49%
Platelets	240 x 10^3^/mcL	150-400 x 10^3^/mcL
Sodium	138 mmol/L	138-145 mmol/L
Potassium	3.9 mmol/L	3.3-4.8 mmol/L
Glucose	92 mg/dL	60-99 mg/dL
Creatinine	0.51 mg/dL	0.59-0.86 mg/dL
Urine protein: creatine	Negative	Negative
Lactic Acid	1.1 mEq/L	0.5-2.2 mEq/L
CRP	3.1 mg/L	0.1-1.7 mg/L
Troponin	< 0.01 ng/mL	< 0.03 ng/mL
TSH	2.28 mcU/mL	0.30-3.0 mcU/mL
Free T4	0.84 mcU/mL	0.66-1.10 ng/dL
D-Dimer	1.89 mcg/mL	0.27-0.49 mcg/mL
Fibrinogen	548 mg/dL	188-450 mg/dL

**Table 2:** Cerebrospinal Fluid Studies CSF: Cerebrospinal Fluid

CSF study	Result
Color	Colorless
Red blood cells	0
Glucose	48
Protein	17
Lactic Acid	1.5
Oligoclonal Bands	None
Cryptococcus	Negative
Bacterial culture	No growth
Fungal culture	No growth

Throughout her admission, multiple services were involved, including obstetrics and gynecology, pediatric infectious disease, pediatric rheumatology, neurology, and physical and occupational therapy. Multisystem inflammatory syndrome in children (MIS-C), autoimmune pathology, and lupus cerebritis was ruled out by each service. She was thought to have suffered from reversible cerebral vasoconstriction syndrome (RCVS), likely leading to her ACA stroke. However, thromboembolism which resolved prior to imaging, could not be ruled out. Her seizure disorder was likely secondary to her stroke. She was extubated in the hospital on day 8. During her recovery, the patient exhibited waxing and waning mentation. She displayed incoherent, delirious, and disinhibited behavior, such as pulling out and ingesting her hair, yelling obscenities at family members and nursing staff, and making hypersexual statements about men outside her room. This behavior was noted to be worse at night. Psychiatry believed her presentation was consistent with delirium secondary to stroke, seizures, and ICU stay. Within several days, her symptoms resolved, and she returned to her baseline mental state. She was ultimately transitioned to inpatient rehabilitation on hospital day 24. She was followed up for six months following her hospitalization. She is reported to experience mild neurologic deficits, such as difficulty writing, but overall has fully recovered.

## Discussion

The medical literature has yet to fully explore post-infectious neurologic complications arising from COVID-19, especially within the pediatric population [2]. A broad differential of post-COVID-19 complications within this population has been slowly gaining attention as the pandemic progresses.

The exact etiology of stroke, in this case, is unknown but likely was due to a combination of pregnancy physiology and acute COVID-19 infection. Pregnancy and the postpartum state are known hypercoagulable states with a risk of thromboembolism fourfold to fivefold increase compared to the non-pregnant state [3]. While the literature regarding COVID-19 infection and the risk of thromboembolism, including stroke, is still evolving, it is estimated that infection may increase risk up to 7.8-fold [1]. 

The mechanism by which COVID-19 causes neurological injury needs to be defined. However, a leading hypothesis theorizes that the coronavirus can bind angiotensin-converting enzyme 2 (ACE2) receptors within the brain, which can then dysregulate blood pressure and affect the blood-brain barrier integrity [4]. Ebrahimpour et al. proposed that new-onset convulsions in the setting of COVID-19 infection may be due to direct infection of the central nervous system by the virus [5]. However, like our case, cerebrospinal fluid studies did not provide evidence of direct COVID-19 CNS infection. These authors also believe that focal neurologic deficits are likely the result of ischemic brain injury inflicted by the inflammatory host response triggered by infection.

In a large cohort study looking at neurological manifestations among patients hospitalized with COVID-19, it was found that there was a 3% incidence of stroke among patients with known neurologic sequelae from COVID-19 and that neurologic syndrome was associated with an increased risk of in-hospital death [1].

## Conclusions

While the respiratory, gastrointestinal, and renal complications of COVID-19 infection are well described, the neurologic sequelae are not as well-characterized. Other exogenous sources can often confound symptoms during a complicated medical admission. It has been well established that pregnant women with COVID-19 are more likely than non-pregnant women to show symptoms and require intensive care, mechanical ventilation, and extracorporeal membrane oxygenation. Pregnant women infected with COVID-19 are also more likely than their non-infected counterparts to experience preterm birth, intrauterine fetal demise, and preeclampsia.

As the COVID-19 pandemic continues, healthcare providers should continue to consider the multifaceted presentation of the disease with consideration of post-infectious presentations. While neurologic sequelae are less well characterized than other significant symptoms associated with COVID-19, it is essential to increase awareness of these symptoms, especially in terms of the management of both maternal and fetal health.
